# Economic comparison of stocker cattle performance on winter wheat vs. summer-dormant tall fescue with nitrogen or interseeded alfalfa in the southern great plains

**DOI:** 10.1093/tas/txae154

**Published:** 2024-11-06

**Authors:** Enoch Adom, Jon T Biermacher, B Wade Brorsen, Sindy M Interrante, Dayton M Lambert, Twain J Butler

**Affiliations:** Department of Agricultural Economics, Oklahoma State University, Stillwater, OK, USA 74078-6026; Department of Agribusiness and Applied Economics, North Dakota State University, Fargo, ND, USA 58108-6050; Department of Agricultural Economics, Oklahoma State University, Stillwater, OK, USA 74078-6026; Noble Research Institute, Ardmore, OK, USA 73401-2124; Department of Agricultural Economics, Oklahoma State University, Stillwater, OK, USA 74078-6026; Bayer Crop Science, St. Louis, MO, USA 63141-7843

**Keywords:** alfalfa, cool-season perennial, cost of gain, economics, grazing, tall fescue, winter wheat

## Abstract

Winter wheat (*Triticum aestivum* L.) is a significant forage source for livestock grazing in the Southern Great Plains (SGP). However, increasing input costs and changing climate conditions compel producers and researchers to search for alternative forage systems, such as cool-season perennials. Specifically, cool-season perennials with summer dormancy traits can survive droughts in the SGP. This paper aimed to determine the net returns of three different types of cool-season perennial summer-dormant tall fescue [*Schedonorus arundinaceus* (Schreb.) Dumort., nom. cons.] systems either with N fertilizer or interseeded with alfalfa (*Medicago sativa* L.) and the traditional graze-out annual winter wheat forage system. The data were from a 5-yr experiment conducted in south-central Oklahoma. Animal performance results indicated that the traditional graze-out winter annual wheat forage system provided more total gains at 434 kg ha^−1^ than the three tall fescue variety systems at 326 kg ha^−1^ (*P* = 0.006) due to more grazing days. Thus, the gross revenue estimated at a cost of gain of $1.60 kg^−1^ gain for wheat at $694 ha^−1^ was greater than the average gross revenue of $521 ha^−1^ for the tall fescue systems. However, the average total cost for the perennial tall fescue systems ($374 ha^−1^) was less than the total cost ($594 ha^−1^) of the wheat system. Overall, the average net returns were similar for all grazing systems at about $145 ha^−1^.

## Teaser

This study demonstrates that cool-season perennial livestock forage systems, particularly when interseeded with legumes, can significantly reduce establishment and production costs compared to traditional annual systems like winter wheat while offering comparable net returns.

## Introduction

Winter wheat can withstand harsh weather conditions (Pinchak et al., 1996) and is a significant contributor to the agricultural economy of the Southern Great Plains (SGP) (Butchee and Edwards, 2013). Winter wheat is particularly important for the livestock sector. An estimated 39% of south-central and eastern Oklahoma wheat acres are used solely for forage (True et al., 2001). In the SGP, stocker cattle typically graze winter wheat from fall to early spring and switch to other grasses in late spring and summer (Coleman and Forbes, 1998). The cost of establishing annual grass forage systems, such as winter wheat, has increased due to rising input prices (fertilizer, fuel) and labor costs (Islam et al., 2011). For instance, between January and December 2021, the USDA-ERS estimated an average annual price increase of 192% for three forms of N fertilizer (anhydrous ammonia, 235%, urea, 149%, and liquid nitrogen. 192%) due to rising natural gas prices (USDA-ERS, 2021). In addition to the growing cost of annual establishment of cereal forage for grazing, poor rainfall between September and December has caused delays in planting winter wheat, causing shortfalls in forage supply during winter and early spring (Malinowski and Pinchak, 2015).

In response to the issues associated with establishing annual forages for grazing, cool-season perennial grasses, such as tall fescue and tall wheatgrass (*Thinopyrum ponticum* Podp.), are being promoted as economical alternatives to annual grasses to reduce production costs and prevent shortfalls in forage supply (Kindiger and Conley, 2002; Hopkins and Alison, 2006; Beck et al., 2008; Coleman et al., 2010; Ren et al., 2022).

Perennial forages such as tall fescue have several agronomic benefits. Tall fescue was introduced into the United States from Europe and has adapted to different soil and climatic conditions (Balasko, 1981). In addition to its high nutritive value (Islam et al., 2011), tall fescue can reduce soil erosion after tillage (Kindiger and Conley, 2002). Researchers have identified and promoted tall fescue varieties with novel nontoxic endophyte (NE) as a solution to fescue toxicity (Gunter and Beck, 2004), which has a significant economic impact due to reduced animal performance. Islam et al. (2011) explored introducing an eastern TF variety with NE in western Oklahoma, comparing annual wheat forage systems with cool-season perennial systems. Their research used a summer-active/continental TF variety, which did not persist because of severe drought conditions. This outcome is similar to other research, which suggests that the acute climate conditions experienced in the SGP may cause traditional cool-season perennial grasses such as ‘Jose’ tall wheatgrass and other cool-season grass species that are summer-active, to fail (Malinowski et al., 2003; Gillen and Berg, 2005).

The previous studies suggest that new research should focus on TF varieties with summer-dormancy traits that withstand warmer temperatures during the cooler months to increase persistence (Malinowski and Pinchak, 2015) by withstanding drought (Craven et al., 2010; Bhamidimarri et al., 2012). Research on the economics of tall fescue varieties with summer-dormancy traits for grazing in Oklahoma has shown promising results. Malinowski (2009) found that summer-dormant tall fescue (SDTF) varieties are well-adapted to the SGP, including Oklahoma, due to their ability to tolerate high temperatures and drought. Malinowski’s findings suggest that these varieties may be a cost-effective option for grazing in the region. However, further research is needed to understand the economic implications of using these varieties compared to other forage options.

Another strategy that could reduce stocker cattle production costs is to plant N-fixing annual legumes (Rouquette and Smith, 2010). The economic benefits of this approach depend on factors such as the legume choice (Brennan and Evans, 2001), management strategies and environmental factors (Peoples et al., 2001), and the price of inorganic fertilizer. This study considers an alfalfa and tall fescue mixture. Lauriault et al. (2005) found that including alfalfa in rotational pastures increased average daily gain (ADG) and TG by 15% and 107%, respectively.

The objective of this research was to determine the performance (ADG, TG, total steer grazing days [TSGD]) and economics (revenues, costs, and net returns) of stocker cattle under three SDTF forage systems (either with synthetic N or alfalfa) and the traditional wheat grazing system common to south-central Oklahoma. The research also evaluates the sensitivity of base-case results to changes in the cost of gain, changes in fertilizer prices, changes in interest rate, and changes in the stand life of alfalfa and tall fescue. The three tall fescue systems included two varieties of SDTF tall fescue fertilized with synthetic N (FLCH-N, ‘Flecha’ and CHSM-N, ‘Chisholm’) and Flecha SDTF interseeded with ‘Bulldog 505’ alfalfa (FLALF). The forage systems considered encompassed two different strategies that have been proposed to improve farmer productivity and profitability: the use of cool-season perennial grasses (Hopkins and Alison, 2006; Beck et al., 2008) and incorporating N-fixing legumes into pastures (Rouquette and Smith, 2010).

Numerous studies have examined the impact of these two strategies on farmer productivity and profits separately. Beck et al. (2008) used data from an experiment on tall fescue (with and without NE), cereal rye (*Secale cereale* L.), and wheat. They concluded that compared with cool-season annual grasses, NE tall fescue can extend the grazing season and decrease the risk of not being able to establish annual forage crops. Similarly, Islam et al. (2011) compared the performance of NE summer-active tall fescue with cereal rye-annual ryegrass (L. multiflorum Lam.) system. They found that factors such as forage mass, nutritive value, and stocker ADG influenced the production and economics of grazing rye-annual ryegrass systems. The annual production cost was lower for tall fescue systems. However, the net return was greater for rye-annual ryegrass systems. A study by Mckee et al. (2017) in the lower transition zone of Alabama also found results comparable to those of Beck et al. (2008) and Islam et al. (2011).

The objectives of the other management strategies have focused on replacing fertilizer N by incorporating N-fixing legumes into pastures. Interrante et al. (2012) evaluated tall fescue systems fertilized with synthetic nitrogen and interseeded with legumes (arrowleaf clover [*Trifolium vesiculosum* Savi], field pea [*Pisum sativum* L.], and hairy vetch [*Vicia villosa* Roth]). The tall fescue systems interseeded with legumes did not perform better than those fertilized with synthetic nitrogen. However, given different weather conditions and/or different legumes (such as alfalfa), this strategy may be a viable replacement option for synthetic N. Lemus et al. (2020) recommended adding at least 28 kg N ha^−1^ in the fall to annual ryegrass interseeded with clover species to help early establishment before the legume contributes biologically fixed N. N fixed in the plant’s stems and leaves are consumed by livestock during grazing and returned to the pasture through dung and urine, making it available for future forage plants. Treatments with legumes only differed marginally ($0.59 ton^−1^ DM forage) from treatments with synthetic N in terms of cost of production.

We found no previous research that examined the relative production and economics of summer-dormant tall fescue compared to wheat pasture grazing systems and/or combining cool-season perennials with alfalfa simultaneously. This study contributes to existing literature. It will also provide farmers with information for determining optimal management practices.

## Materials and Methods

### Data and Experimental Description

All animal procedures in the following study were conducted in accordance with the Guide for the Care and Use of Agricultural Animals in Research and Teaching and received approval from the Noble Research Institute’s Animal Care and Use Committee (IACUC) before the study began in 2013.

The 5-yr study (2013/14 to 2017/18) included three replications of a completely randomized design grazing experiment, which started in the fall of 2013 at the Noble Research Institute’s Headquarter Research Farm near the community of Ardmore in south-central Oklahoma, USA (34°11ʹ11.3″N 97°05ʹ36.1″W). The soil type of the study area is mainly Heiden clay loam (fine, montmorillonitic, thermic Udic Chromusterts) with an average pH of 7.1 and organic matter of 32 g kg^−1^. The study used 0.81-hectare grazing paddock for each of the three grazing systems, including (1) the conventional system of graze-out annual ‘NF101’ winter wheat, (2) summer-dormant tall fescue varieties ‘Flecha’ with N fertilization (FLCH-N) and’Chisholm’ with N fertilization (CHSM-N); and (3) FLCH with interseeded ‘Bulldog 505’ alfalfa (FLALF) in alternating and perpendicular orientation (Butler et al., 2011). Steers with 5-yr average initial body weight (BW)s of 278 kg (BW = 270 ± 29,286 ± 37,296 ± 55,217 ± 11, and 297 ± 15 kg for the respective growing seasons from 2013 to 2018) were weighed every 28 d.

### Agronomic Practices

Study site preparation was initiated in May 2013 by applying glyphosate (N-(phosphonomethyl)-glycine), dicamba (3,6-dichloro-2-methoxybenzoic acid) and 2,4-D (2,4 dichlorophenoxyacetic acid) at an average rate of 1.9 kg a.i. ha^−1^, 0.16 kg a.i. ha^−1^, and 1.17 kg a.i. ha^−1^, respectively, to reduce weed species. Seedbeds for all paddocks and systems were prepared using tillage, beginning with chisel plowing in late July, followed by tandem discing and offset discing, and finished with field cultivation in August (John Deere, Moline, IL). Paddocks for the wheat system were tilled only in the first year, with no-till adopted in subsequent years. Paddocks were fertilized each year with P_2_O_5_ based on soil test recommendations (Zhang et al., 2009) using either diammonium phosphate (18-46-0) or triple super phosphate (0-46-0) between the rates of about 25.9 kg P ha^−1^ (56 kg P_2_O_5_ ha^−1^) to 103.1 kg P ha^−1^ (224 kg P_2_O_5_ ha^−1^). Paddocks for the forage systems with synthetic N (wheat, FLCH-N, and CHSM-N) were fertilized with urea (46-0-0) such that the total N in the soil (fertilizer N plus soil residual N) was 112 kg N ha^−1^ based on soil test results. Winter wheat was planted in the first year using a grain drill. In subsequent years, winter wheat was planted each year using a no-till drill in mid-September (2014 to 2017) at a rate of 95 kg ha^−1^ of pure live seed (PLS). Tall fescue in the FLCH-N and CHSM-N systems was planted once in mid-September of 2013 at a rate of 17 kg ha^−1^ PLS. In year two, additional tall fescue seeding occurred to thicken the stand in some paddocks. In the FLALF system, tall fescue and alfalfa were planted in mid-September of 2013 at a rate of 17 and 13 kg of PLS ha^−1^, respectively. However, the stand was lost after the end of the second year due to excessive (300 mm rainfall in 24 h); hence, the tall fescue and alfalfa were replanted in September 2015.

### Animal Management

Black Angus stocker steers (*Bos taurus*, 276 ± 42 kg initial BW) were procured in early October from the Oklahoma National Stockyards in Oklahoma City. Animals were preconditioned for a minimum of 45 d prior to grazing. Each year, within 24 h of receiving the animals, all steers were subjected to the same preconditioning process outlined by Moore et al. (2021). Cattle were implanted with a growth hormone (Revalor G; Merck Animal Health), given a vaccination for bovine rhinotracheitis-virus and diarrhea-parainfluenzerespiratory syncytial virus (BoviShield Gold 5; Zoetis Inc.), administered clostridium chauvoei-seticum-haemolyticum-novyi-sordelliitetani-perfringens types C&D bacterin-toxoid (Calvary 9; Merck Animal Health) for protection against bacterial infections, provided treatment for foot rot (Fusogard; Elanco US Inc., Farm Animal Business), and administered Mannheimia haemolytica and *Pasteurella multocida* bacterin (PMH IN, Merck Animal Health) to prevent respiratory infection. All steers were dewormed (Valbazen, Zoetis Inc.) and received treatment for fly and lice control (CyLence; Bayer Healthcare LLC). The mortality rate throughout the study period was less than 1%.

### Forage Management

Paddocks were stocked using a variable stocking rate method (Bransby, 1989). Each experimental unit was randomly assigned two tester steers. Later, grazers were added to adjust stocking rates based on forage mass to achieve the target forage allowance (0.8 kg forage per kg animal live weight). Forage allowance levels used to set the stocking rates for testers and grazers were set following guidance from methods outlined by Sollenberger et al., 2001 and Zhang et al., 2008. Grazing was initiated yearly when forage mass was at least 2.0 Mg DM ha^−1^. Grazing was terminated when forage mass reached 1.0 Mg DM ha^−1^, or when the nutritive value of forage became low to a point where it limited animal gains (Redmon et al., 1995; Beck et al., 2008).

Measurements of animal performance were computed for each forage system on a per-hectare basis. The ADG, average on-weights and off-weights, and TSGD were determined for the tester steers for each paddock, year, and system. The ADG of tester steers was calculated as the difference between the final BW and the initial BW of each steer, divided by the total number of grazing days. Total grazing days for each paddock in each year were computed as the sum of the grazing duration for each steer (both testers and grazers) that grazed in that paddock during that year. The total grazing days (from testers and grazers) were multiplied by the ADG (from testers) to calculate each system’s total gain per hectare. Finally, data were collected on TSGD, TG for each forage system. Subsequently, the collected data were used to calculate the ADG. The outline of production activities is reported chronologically by month and forage system in Table 1.

**Table 1.** Chronology of 4-yr average forage production activities by month and system

Production activity	Month	WHEAT	FLCH-N	CHSM-N	FLALF
Primary tillage—chisel plowing	Jul–Aug	x	x	x	x
Secondary tillage—offset and tandem discing	Jul–Aug	x	x	x	x
Seedbed preparation/field cultivation	Aug	x	x	x	x
Apply N, P, K, and S fertilizers	Aug	x	x	x	x
Plant wheat, alfalfa and tall fescue	Sept	x	x	x	x
Apply insecticide to control armyworm	Sept	x	-	-	-
Apply nitrogen fertilizer (topdress)	Jan–Feb	x	x	x	x
Apply herbicides to control broadleaf weeds	Feb–Mar	x	-	-	-
Apply insecticide to control weevil and looper	Mar	-	-	-	x
Apply herbicide to control broadleaf weeds	Jun		x		
Offset discing to incorporate wheat stubble	June	x	-	-	-

### Economic Analysis

Enterprise budgeting was used to determine gross revenue, specified costs, and net return for the four forage grazing systems (AAEA, 2000). Gross revenue for each forage system was calculated as the product of average accumulated TG (kg ha^−1^) and the region’s average winter wheat pasture cost of gain. It is a common practice in the region for livestock owners to lease pastureland from wheat producers and make payments based on the net live weight gain attributed to the wheat pasture (True et al., 2001; Hossain et al., 2004; Varner et al., 2012). The cost of gain charged is based on wheat grain price forecasts and generally remains unchanged, even if cattle prices fluctuate beyond the initial expectations. In the base-case scenario, an average cost of gain of $1.60 kg^−1^ gain based on Varner et al. (2012) was used to calculate and compare gross revenue among the systems.

The main costs for each system were steer preconditioning costs, land preparation costs, seed and seed establishment costs, prorated annual fixed costs for tall fescue and alfalfa, fertilizer costs, herbicide costs, and the interest cost for owning steers during the grazing period. Interest for all other operating expenses was also included. The cost for each input for each system is the product of the 4-yr average quantity of each input multiplied by the expected input price for each input. Total cost is the sum of all individual input costs.

Following Moore et al. (2021), a 2020 average cost of $2.02 hd/d was applied to cover expenses associated with pharmaceutical/healthcare treatments, hay, feed, and minerals incurred during preconditioning. The expected regional market prices of $1.82, $1.52, and $1.54 kg^−1^ were used for urea (46-0-0), UAS (33.5-0-0-12), and DAP (18-46-0) fertilizers, respectively, indicating the $N\text{-}P_{2}O_{5}-K_{2}O-S$ composition. The expected seed prices used were $17.64 kg^−1^ (alfalfa), $0.71 kg^−1^ (wheat), and $12.72 kg^−1^ (tall fescue). The average expected prices of insecticides and herbicides were $25.68 and 22.97 L^−1^, respectively. Custom rates for machinery operations were used in the analysis (Sahs, 2022). The custom rate for spraying insecticide was $14.92 ha^−1^, and the custom rate for applying liquid herbicide was $16.20 ha^−1^. The custom rate of $14.70 ha^−1^ was used for fertilizer applications. Land and seedbed preparation rates included chisel plowing ($36.69 ha^−1^), offset discing ($36.83 ha^−1^), field cultivation ($26.56 ha^−1^), and drilling (average) ($34.30 ha^−1^). An annual interest rate of 9% was used to calculate the opportunity cost of capital during the growing season. The expected life of tall fescue pasture was assumed to be 10 yr for the tall fescue forage systems, and the expected life of alfalfa for the FLALF system was assumed to be 5 yr. In this study, longer life expectancies are used for the tall fescue and alfalfa stands compared to previous studies at the same site (e.g., Islam et al., 2011), which utilized summer-active tall fescue, because the SDTF used in this study has now lasted more than 7 yr, including surviving drought in 2021 and 2022. Similarly, 5-yr life span is used for alfalfa because the stands of tall fescue interseeded with alfalfa survived for 5 yr before diminishing to the point of needing replacement. The annual fixed cost of establishment of tall fescue and alfalfa for their respective systems was calculated assuming the total establishment cost was amortized over the expected stand life at an interest rate of 9%. The budget includes the expense of additional supplemental overseeding of tall fescue in the FLCH-N, and CHSM-N systems. However, it does not cover the cost of replanting tall fescue and alfalfa in the FLALF system after the second year, as this is assumed to be unusual. The consequence of re-establishment could lead to potential yield losses.

The net returns for each treatment were calculated by subtracting the total cost of production from gross revenue, which results in net returns to land, management, and farm overhead.

Sensitivity analysis was performed to assess the robustness of the net returns in the base-case scenario to changes in fertilizer prices (urea, DAP, UAS), changes in the cost of gain, changes in the annual interest rate, and changes in the stand life of tall fescue and alfalfa, all other factors held constant. The sensitivity of the results was also evaluated with respect to the inclusion of the cost of replanting tall fescue and alfalfa in the FLALF system after the second year. Varying the pasture cost of gain, fertilizer prices, interest rates, and stand life ensures the robustness of the results and conclusions across various market and growing conditions.

### Statistical Analysis

The data were analyzed using the MIXED procedure in SAS (SAS Institute Inc, 2014). The effect of the forage system on animal performance (TG, ADG), economic measures (total cost, gross revenue, and net return), and TSGD were estimated using analysis of variance (ANOVA). The forage system treatments entered the statistical model as fixed effects, while production years entered the model as random effects. Pairwise comparisons of the least squares means of the systems were performed using the PDIFF function in SAS. The PDIFF procedure uses Fisher’s least significant difference method. An F-test was used to determine statistical differences between forage systems at the 5% significance level.

The data-generating process is represented mathematically as


$$\begin{array}{l} y_{it}=\mu_{i}+\tau_{t}+\epsilon_{it} \end{array}$$ (1)

where $y_{it}$ is either the animal performance indicator (ADG, TGD, and TG) or the economic measure (total cost, gross revenue, and net returns) from the system i, in year t(t=1, …, 4), $\mu_{i}$ is the forage system i ’s mean, $\tau_{t}$ is the year random effect distributed as $\tau_{t}∼N(0,\sigma_{t}^{2})$, and $\epsilon_{it}$ is an independent and identically distributed error term with $\epsilon_{it}∼N(0,\;\sigma_{\epsilon }^{2})$.

The null hypothesis of no-year random effects was rejected at the 95% confidence level using a likelihood ratio test (Greene, 2002) for all three animal performance measures and total cost. Heteroscedasticity may exist because the observations are averages across replications and because the number of animals assigned in each replication was unequal. Thus, to correct for any potential heteroscedasticity, maximum likelihood was used to estimate all the models, as suggested by Richter and Brorsen (2006).

## Results and Discussion

### Animal Performance

**Figure 1.**
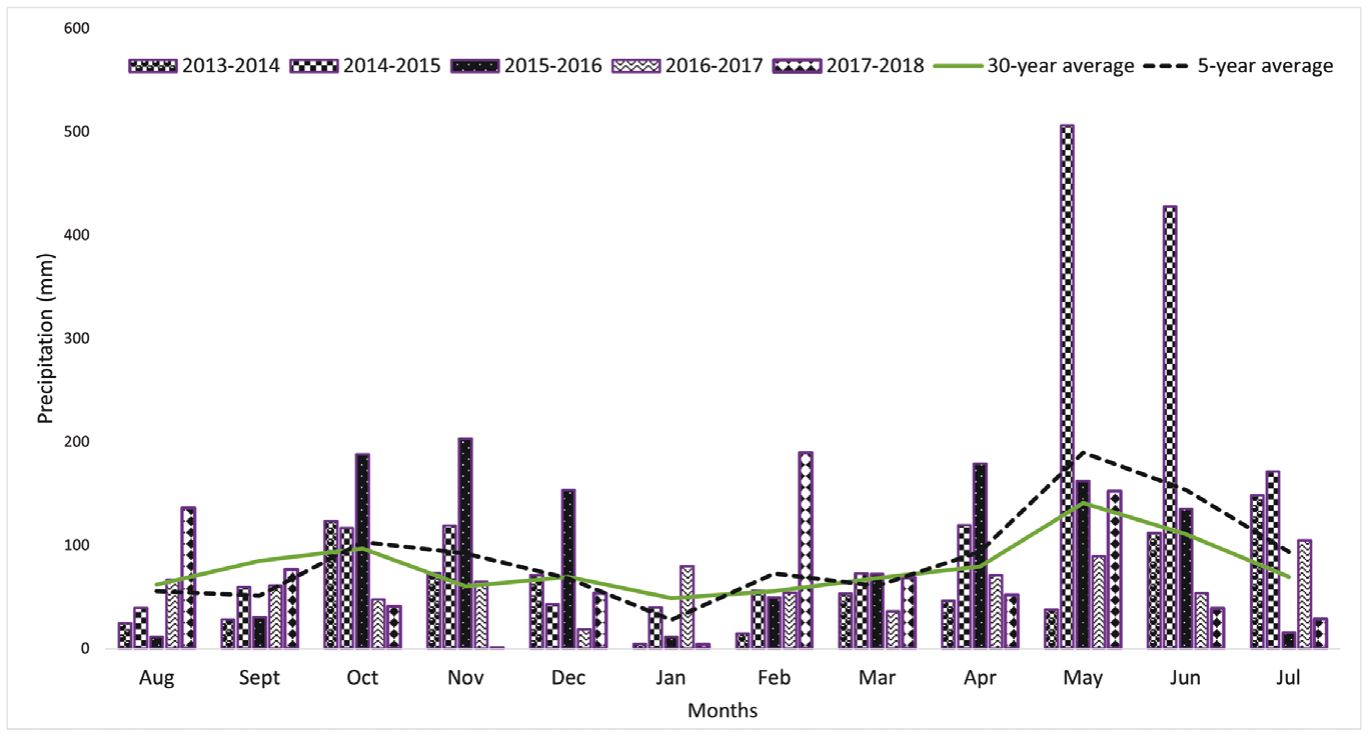
Monthly precipitation (mm) for growing seasons at Ardmore, Oklahoma. Source: www.mesonet.org

Figure 1 illustrates the precipitation distribution for the five growing seasons. The data indicated that precipitation during the establishment months (August to November) is closely aligned with the 30-yr average (76 mm) precipitation. The 2016 to 2017 growing season had the lowest precipitation, about 26% lower than the 30-yr average, while precipitation was highest in the 2014 to 2015 growing season, about 87% higher than the 30-yr average.

Table 2 reports the least squares means for grazing initiation and termination weights and dates, ADG, TSGD, and total gain. There were more animal grazing days ha^−1^ associated with wheat, FLCH-N, and CHSM-N systems than FLALF (*P* = 0.0004). Pairwise comparisons indicated that both tall fescue systems (CHSM-N and FLCH-N) were not different from the traditional wheat system regarding the number of grazing days (*P* = 0.5588). However, wheat had more grazing days than FLALF (*P* = 0.0011). The lesser TSGD observed in the FLALF system is attributed to competition between alfalfa and tall fescue (Interrante et al., 2012). In comparison, TSGD for FLCH-N (450 d ha^−1^) was greater than the values reported by Islam et al. (2011) (385 d ha^−1^) and Interrante et al. (2012) (429 d ha^−1^).

**Table 2.** Least squares means of 5-yr annual measures of animal performance, gross revenues, costs, and net returns to land, labor, and management by forage system

	Forage System				
Item	WHEAT	FLCH-N	CHSM-N	FLALF	*P*-value
**Animal performance**					
Grazing initiation date	Dec. 16	Dec. 01	Nov. 30	Jan. 13	
Grazing initiation weight, kg	283A	267B	251C	293A	<0.0001
Grazing termination date	Apr. 20	May. 19	May. 21	May. 18	
Grazing termination weight, kg	365A	350B	332C	337C	<0.0001
Average daily gain, kg d^−1^	0.97A	0.80B	0.79B	1.0A	0.1072
Stocking rate, head ha^−1^	1.40A	1.22AB	1.07B	1.88C	<0.0001
Total steer grazing days (head days, ha^−1^)	462A	450A	482A	371B	0.0004
Total gain, kg ha^−1^	434A	332B	316BC	329C	0.0064
**Economics**					
Tillage and seedbed preparation costs, $ ha^−1^	100	127	127	110	
Cost seeds and custom drill, $ ha^−1^	53	293	359	482	
Herbicides applied during establishment year, $ ha^−1^	85	116	116	116	
Fertilizers applied during establishment year, $ ha^−1^	193	0	0	123	
Total forage establishment cost, $ ha^−1^	341	536	602	831	
Establishment costs amortized at 9% APR	—	84	94	173	
Forage maintenance cost, $ ha^−1^	103	215	211	128	
Interest for steer ownership, $ ha^−1^	105	108	116	89	
Interest on operating costs, $ ha^−1^	4.62	9.66	9.49	5.73	
Total cost, $ ha^−1^	549A	394A	376B	352B	< 0.0001
Gross revenue, $ ha^−1^	694A	532B	506B	526B	0.0060
Net returns, $ ha^−1^	144A	137A	128A	174A	0.7957

WHEAT = Annual ‘NF101’ winter wheat fertilized with 112 kg N ha^−1^ nitrogen, FLALF = ‘Flecha’ tall fescue interseeded with ‘Bulldog 505’ alfalfa, FLCH-N = Flecha tall fescue fertilized with 112 kg N ha^-1^ nitrogen, and CHSM-N = ‘Chisholm’ (NFTF1700) tall fescue fertilized with 112 kg N ha^−1^ nitrogen. Means in the same row denoted with the same uppercase letter are not different from each other using Fisher’s least significant difference method in SAS PROC MIXED procedure (5% significance level). Forage maintenance costs include cost of herbicide, fertilizer, and insecticide after establishment.

There were no differences in ADG between the forage systems (*P* = 0.1072, Table 2). The ADGs were greater than Interrante et al. (2012) reported for a tall fescue fertilized with N forage system (0.69 kg d^−1^). Islam et al. (2011) reported an average ADG of 0.93 kg d^−1^ for an NE tall fescue, which is greater than the average ADG for FLCH-N and CHSM-N systems but lesser than that of FLALF. Similarly, Reuter and Horn (2002) reported an average ADG of 0.95 kg d^−1^ for steers grazing three different types of cool-season perennial grasses. A pairwise comparison of the ADG results shows that the wheat system had greater ADG (25% to 26%) than FLCH-N and CHSM-N (*P* ≤ 0.0281). Although the FLALF system had the fewest grazing days, the mean ADG was similar to the mean ADG of the wheat system (*P* = 0.6693).

Total gain differed across the systems (Table 2, *P* = 0.0064) and was greatest in the wheat system (434 kg ha^−1^). Mckee et al. (2017) reported similar total gains of 341 kg ha^−1^ for tall fescue with an inorganic N system and 388 kg ha^−1^ for tall fescue with a legume system. The average total gain of 326 kg ha^−1^ for the perennial systems (FLCH-N, CHSM-N, and FLALF) was higher than the average total gain of 283 kg ha^−1^ reported by Reuter and Horn (2002) for steers grazing three types of cool-season perennial grasses. Pairwise comparisons indicated that wheat had a significantly greater TG than all other systems (*P* ≤ 0.0068).

### Economics

Average production costs, gross revenue, and net return to land, labor, and management for each forage system are reported in Table 2. Total costs, which comprise prorated establishment costs of perennial systems, differed among systems (*P* < 0.0001; Table 2), with the greatest estimated total cost being associated with the wheat system at $549 ha^−1^ (calculated as the sum of the average annual establishment cost [$331 ha^−1^], maintenance cost $103, interest expense for steer ownership [$105 ha], and interest on operating capital [$4.62 ha^−1^]). Pairwise comparisons showed that the total cost for wheat was significantly higher than the total costs for the perennial systems (*P* < 0.0001). However, the total costs for the tall fescue systems (FLCH-N, CHSM-N, and FLALF) were not significantly different from each other (*P* ≥ 0.0658). The average total cost for the perennial systems (FLALF, FLCH-N, and CHSM-N) is about $172 ha^-1^ less than the traditional wheat system cost. The average gross revenue per hectare generated by the wheat system is about 30.5%, 32%, and 37%, respectively, more than revenue from the FLCH-N, FLALF, and CHSM-N systems, respectively, at an average cost of gain of $1.6 kg^−1^ gain. This result corresponds with additional revenue of about $162, $168, and $188, respectively, per hectare compared to FLCH-N, FLALF, and CHSM-N, respectively (Table 2).

The adoption of a forage species or forage system by stakeholders depends on the sustainability and stand maintenance of the forage and the potential profitability of management strategies. The net revenue analysis for the base-case scenario in Table 2 provides insight into the economic performance of the forage systems. There were no differences in net returns between the forage systems (*P* ≥ 0.7957). Even though net revenues were not different, the tall fescue systems offer opportunities to supplement the wheat system for year-round forage production, reducing the risk of forage supply shortages during winter and early spring.

Several studies examined various forage systems’ economic viability and performance, including traditional annual and cool-season perennial systems. A study by Islam et al. (2011) indicated that the rye-annual ryegrass system exhibited greater net returns ($279 ha^−1^) compared to the tall fescue system ($217 ha^−1^), which is different from the results of this study in which the annual wheat and perennial systems had similar net returns. However, Beck et al. (2008) reported a net profit of $136 ha^−1^ for annual ryegrass and $219 ha^−1^ for tall fescue with a NE grazing system but did not include the establishment cost.

In a study by Interrante et al. (2012), the economic analysis of steers grazing tall fescue with annual legumes or fertilized with nitrogen (TFN) demonstrated that TFN had a greater net return ($224 ha^−1^) than grazing tall fescue with annual legumes ($93 ha^−1^). The difference in net return was due to the TFN system’s greater number of grazing days, leading to greater TG per hectare. This study reports a different result: the FLALF system yielded similar net returns as the tall fescue systems fertilized with synthetic N (FLCH-N and CHSM-N). This finding suggests that incorporating alfalfa into the tall fescue system (FLALF) can provide comparable economic benefits to traditional nitrogen-fertilized tall fescue systems (FLCH-N and CHSM-N). This highlights the potential of FLALF as a viable alternative that may reduce reliance on synthetic fertilizers while maintaining profitability.

Table 3 presents the results of the sensitivity analysis comparing the net returns for all the systems to changes in the prices of fertilizers (DAP, UAS, and urea), cost of gain, interest rate, and tall fescue and alfalfa stand lives. The results indicated that the net returns for the wheat system decreased by 26%, 53%, 78%, and 104%, respectively, for fertilizer price increments of 25%, 50%, 75%, and 100%, respectively. Similarly, the FLCH-N and CHSM-N net returns declined between 21% and 87% and 23% and 91%, respectively. In contrast, the net returns for FLALF experienced smaller reductions, decreasing by 1%, 2%, 4%, and 5%, respectively, for fertilizer price increments of 25%, 50%, 75%, and 100%, respectively, reflecting its relative resilience to changes in fertilizer prices since this system did not require N for establishment. On average, incremental changes in fertilizer prices caused the expected net returns for WHEAT, FLCH-N, CHSM-N, and FLALF to decrease to $68, $77, $69, and $170 ha^−1^ respectively. Additionally, when fertilizer prices were reduced by 25% and 50%, respectively, the net returns for all systems increased, with the net returns for the wheat system increasing by 25% and 51%, respectively, and FLALF increasing only by 2%, and 3%, respectively. This result suggests that the FLALF system may experience less impact from changes in fertilizer prices compared to other systems, due to its reduced reliance on nitrogen for establishment.

**Table 3.** Changes in expected net return (US$/ha) relative to incremental adjustments in fertilizer prices, pasture cost of gain, and stands life across forage systems

			Forage system net return, $ ha^−1^				
Input	Scenario	Price/year	WHEAT	FLCH-N	CHSM-N	FLALF	*P-value*
	Base-case	0.83 to 0.69 to 0.70	144A	137A	128A	174A	0.7957
Fertilizer, $ kg^−1^ (Urea-UAS-DAP)	25% increase	1.04 to 0.86 to 0.88	106A	108A	99A	172B	0.3720
	50% increase	1.25 to 1.04 to 1.05	68A	77A	69A	170B	0.0875
	75% increase	1.45 to 1.21 to 1.23	31A	47A	40A	168B	0.0128
	100% increase	1.66 to 1.38 to 1.40	-6A	18A	12A	166B	0.0012
	25% decrease	0.62 to 0.52 to 0.53	180A	167A	157A	178A	0.9785
	50% decrease	0.42 to 0.35 to 0.35	218A	197A	186A	179A	0.8695
Cost of gain, $ kg^−1^ gain	15% decrease	1.36	40A	57A	52A	95A	0.5356
	30% decrease	1.12	−63A	−23AB	−24AB	16B	0.1437
	15% increase	1.84	247A	217A	205A	253A	0.8415
	30% increase	2.08	351A	297A	281A	332A	0.7792
Interest rate	decrease 9 to 5%	5%	195A	200A	192A	232A	0.8191
	increase 9 to 12%	12%	105A	90A	79A	130A	0.7581
Stand lost	Inclusion of second establishment cost		149A	145A	140A	107A	0.7854
Tall fescue and alfalfa stand life (years)	5 to 3 years	5 to 3	144A	84A	71A	99A	0.6017

WHEAT = Annual ‘NF101’ winter wheat fertilized with 112 kg N ha^−1^ nitrogen, FLALF = ‘Flecha’ tall fescue interseeded with ‘Bulldog 505’ alfalfa, FLCH-N = Flecha summer-dormant tall fescue fertilized with 112 kg N ha^−1^ nitrogen, and CHSM-N = ‘Chisholm’ (NFTF1700) summer-dormant tall fescue variety fertilized with 112 kg N ha^−1^.

Holding constant other factors, a 15% decline in the cost of gain ($1.36 kg^−1^ gain) generated substantial declines in net returns for all systems but were not significantly different (*P =* 0.5356). The system with the greatest decline in net return was the wheat system (−$108), while the perennial systems experienced, on average, a $78 decline in net returns. Similarly, as the cost of gain increased to $1.84 kg^−1^ gain, there was a substantial increase in the net returns across the systems, but they were not significantly different (*P* = 0.8415). The estimated net return was greatest in FLALF ($253 ha^−1^), followed by wheat ($247 ha^−1^), FLCH-N ($217 ha^−1^), and CHSM-N ($205 ha^−1^), respectively. The result indicated that given a 15% to 30% decrease or increase in cost of gain, the average net returns from the perennial systems were still comparable to the net return from the traditional annual system.

The sensitivity on how changes in the stand life of tall fescue from 10 to 5 yr and the stand life of alfalfa from 5 to 3 yr impact the net returns for each system (Table 3). The decline in the stand life negatively affects the net returns of the perennial systems. The net returns of FLCH-N, CHSM-N, and FLALF decreased to $84, $71, and $99 ha^−1^, respectively. On average, the net return of the traditional annual system was about $60 ha^−1^ greater than the perennial systems when the stand life of tall fescue and alfalfa is assumed to be 5 and 3 yr, respectively. Similarly, the inclusion of the cost of replanting tall fescue and alfalfa in the FLALF system after the second year reduced the net return for the FLALF system by 38%. Finally, the sensitivity analysis with respect to changes in interest rates showed that a decrease in the interest rate to 5% resulted in increased net returns across all forage systems, with the FLALF system showing the highest return of $232 ha^−1^, while an increase to 12% significantly reduced net returns, particularly for the WHEAT system, which dropped to $105 ha^−1^.

## Conclusions

This paper aimed to determine the effects of three different types of tall fescue forage systems and a traditional annual winter wheat forage system on animal performance and economic variables. Although the TSGD were not significantly different between the wheat system and the nitrogen-fertilized tall fescue systems (FLCH-N and CHSM-N), the wheat system yielded greater TG compared to all the other systems. This difference in TG is likely due to the marginally higher ADG observed in the wheat system compared to the perennial systems, even though the differences in ADG were not statistically significant. The average ADG, and TSGD reported were comparable to those reported by other studies (Reuter and Horn, 2002; Islam et al., 2011; 2012; Mckee et al., 2017).

The average gross revenue per hectare generated by the wheat system was about 33% greater than revenue from the perennial systems. However, the perennial tall fescue systems produced significant savings from reduced establishment costs. Consequently, the average net returns from the tall fescue systems were comparable to those from the traditional annual wheat forage system.

The sensitivity analysis indicated that incremental changes in fertilizer prices did not alter the results. Specifically, wheat, FLCH-N, and CHSM-N exhibited comparable net returns. In contrast, FLALF displayed a notably greater net return than the rest of the systems as fertilizer prices increased. Similarly, the sensitivity analysis of a 15% decrease or increase in the cost of gain produced average net returns for the perennial systems that were comparable to the net return from the traditional annual system. Finally, the sensitivity analysis concerning the life stands of alfalfa and tall fescue shows that the traditional wheat systems may be preferred to all three tall fescue systems if the stand life of tall fescue and alfalfa is reduced to 5 and 3 yr, respectively.

Based on these findings, even though the economic performance of the tall fescue perennial forage systems does not outweigh that of the traditional annual winter wheat system, there still exists a potential for the perennial systems to supplement the wheat system for year-round forage production, reducing the risk of forage supply shortage during winter and early spring. In addition, tall fescue systems have other environmental benefits, such as improving soil organic matter, reducing soil nutrient loss, and moderating soil erosion (Kindiger and Conley, 2002).
